# News Items

**Published:** 1919-03

**Authors:** 


### NEWS ITEMS.

### The Harrison Act.

As amended by the new War Revenue Act, will be mailed postpaid to any druggist, physician, dentist or veterinarian who will send a postal request therefor to ''Mailing Department, Parke, Davis & Co., Detroit, Mich.'' Please observe directions strictly.

Physicians, dentists, druggists and manufacturers or dealers who sell or administer narcotic drugs are required under the new revenue bill to register and pay license taxes to revenue collectors within the next thirty days.

This amendment of the Harrison narcotic drug act also taxes opium, cocaine and derivatives or medicines containing these at the rate of one cent an ounce and makes it illegal to sell drugs not bearing revenue stamps.

Physicians, dentists, veterinary surgeons and other practitioners are taxed $3 a year for the drug privilege, but they will be credited with the $1 tax under the old law. Druggists and other retail dealers are taxed $6 a year, wholesalers $12, and manufacturers or importers $25.

---

A curious feature in connection with the epidemic of influenza in South Africa, where a total of 50,000 deaths has left no fewer than 2,000 orphans in Capetown alone, is that the disease has spread to the baboons in the outlying districts and they are reported to by dying ''like flies.''

---

The virus of trench fever and that of influenza and of some forms of nephritis have been isolated and identified, according to a report submitted to the director general of the army medical service, in France by a number of army medical officers who have been investigating the causes of these diseases.

According to this official statement, the virus in each case has been proved to be a minute globular cell, varying in size and behavior in three types of disease. Investigations which have been conducted have resulted, it is believed, in the isolation of the germs of mumps, measles and typhus, the causes of which have hitherto been obscure and the baccili of which have never before been isolated.

